# Turnover of C99 is Controlled by a Crosstalk between ERAD and Ubiquitin-Independent Lysosomal Degradation in Human Neuroglioma Cells

**DOI:** 10.1371/journal.pone.0083096

**Published:** 2013-12-20

**Authors:** Hianara A. Bustamante, Andrés Rivera-Dictter, Viviana A. Cavieres, Vanessa C. Muñoz, Alexis González, Yimo Lin, Gonzalo A. Mardones, Patricia V. Burgos

**Affiliations:** Department of Physiology, School of Medicine and Centro de Investigación Sur-Austral en Enfermedades del Sistema Nervioso, Universidad Austral de Chile, Valdivia, Chile; Torrey Pines Institute for Molecular Studies, United States of America

## Abstract

Alzheimer’s disease (AD) is characterized by the buildup of amyloid-β peptides (Aβ) aggregates derived from proteolytic processing of the β-amyloid precursor protein (APP). Amyloidogenic cleavage of APP by β-secretase/BACE1 generates the C-terminal fragment C99/CTFβ that can be subsequently cleaved by γ-secretase to produce Aβ. Growing evidence indicates that high levels of C99/CTFβ are determinant for AD. Although it has been postulated that γ-secretase-independent pathways must control C99/CTFβ levels, the contribution of organelles with degradative functions, such as the endoplasmic reticulum (ER) or lysosomes, is unclear. In this report, we investigated the turnover and amyloidogenic processing of C99/CTFβ in human H4 neuroglioma cells, and found that C99/CTFβ is localized at the Golgi apparatus in contrast to APP, which is mostly found in endosomes. Conditions that localized C99/CTFβ to the ER resulted in its degradation in a proteasome-dependent manner that first required polyubiquitination, consistent with an active role of the ER associated degradation (ERAD) in this process. Furthermore, when proteasomal activity was inhibited C99/CTFβ was degraded in a chloroquine (CQ)-sensitive compartment, implicating lysosomes as alternative sites for its degradation. Our results highlight a crosstalk between degradation pathways within the ER and lysosomes to avoid protein accumulation and toxicity.

## Introduction

Alzheimer’s disease (AD) is characterized by the accumulation of aggregated amyloid-β (Aβ) peptide species derived from successive proteolytic cleavages of the β-amyloid precursor protein (APP) [1]. The action of β-secretase (also called BACE1) produces a carboxy-terminal fragment-β (C99; also called CTFβ) [2], which is subsequently cleaved by γ-secretase to release Aβ [3]. Proteolytic cleavage by γ-secretase is regulated by substrate availability, with high levels of C99 increasing the probability of γ-secretase cleavage and Aβ generation [4], [5], [6]. Several reports have postulated that C99 levels are regulated by γ-secretase-independent pathways [4], [5], [6], [7], [8], [9]; however, the contribution of these degradation pathways, such as those working in the endoplasmic reticulum (ER) or in lysosomes, to the turnover of C99 and Aβ production is still unclear. The endoplasmic reticulum (ER) has a key role in protein quality control and degradation in coordination with the proteasome [10]. Proteins failing to fold after several attempts can be translocated across the ER membrane back to the cytosol for their degradation through a ubiquitin-dependent proteasome system, a process collectively termed ER-associated degradation (ERAD) [11]. Several reports have demonstrated that Aβ_42_, the most toxic form of Aβ, is generated within the ER, strongly suggesting that C99 must be generated to some extent within this compartment [12], [13], [14]. Indeed, accumulation of APP at the ER results in the production of the N-terminal soluble fragment generated by β-secretase [15]. Moreover, mutations in the AD-linked genes PS1 and PS2 that selectively increase the production of Aβ_42_ strongly accumulate C-terminal fragments within the ER and the Golgi apparatus [16], [17]. In this report, we investigated the turnover and amyloidogenic processing of C99 in human H4 neuroglioma cells stably expressing a GFP-tagged C99 construct in which we introduced substitutions that abolished its non-amyloidogenic proteolytic processing by α-secretase [18], and cleavage by caspase activity [19]. We observed that C99 is localized largely at the Golgi, a different distribution compared to full-length APP, which is predominantly localized in endosomes [19], [20], [21]. We found that C99 is actively degraded at the ER in an ubiquitin and proteasome dependent manner, requiring polyubiquitination of its cytosolic lysine residues. Furthermore, we observed that inhibition of the early degradation of C99 at the ER enhances its degradation within acidic compartments, and when both degradation pathways are impaired C99 accumulates at the cell surface. Finally, we observed that degradation of C99 within acidic compartments, in response to proteasome inhibition, was not dependent on its cytosolic lysine residues, indicating that C99 is degraded in lysosomes in a ubiquitin-independent manner. Unexpectedly, we found that delivery of C99 to the plasma membrane was diminished in the absence of cytosolic lysine residues, instead resulting in a strong accumulation of C99 at the Golgi apparatus, suggesting that ubiquitination mediates its trafficking to the cell surface. Altogether, we propose that C99 can be generated within the ER where it can be efficiently degraded by ERAD. If this process is diminished C99 can be degraded instead within lysosomes in a ubiquitin-independent manner, highlighting a crosstalk mechanism between two degradative organelles that might modulate the production of Aβ species.

## Materials and Methods

### Chemical Reagents and Antibodies

The proteasome inhibitor MG132, and *N*-ethylmaleimide were from Calbiochem (Merck Millipore). Chloroquine (CQ), cycloheximide (CHX), Brefeldin A (BFA), *N*-[(3,5-difluorophenyl)acetyl]-l-alanyl-2-phenyl]glycine-1,1-dimethylethyl ester (DAPT), dithiothreitol (DTT), a cocktail of protease inhibitors, iodoacetamide and chloramphenicol were from Sigma-Aldrich (St. Louis, MO). The following mouse monoclonal antibodies were used: 6E10 to APP (Covance Inc.); WO2 to APP (Merck Millipore); horseradish peroxidase (HRP)-conjugated anti-HA (Macs Miltenyi Biotec); and the clone AC-74 to β-Actin (Sigma-Aldrich). The rabbit serum to GFP was kindly provided by R. Hegde (MRC Laboratory of Molecular Biology, Cambridge, UK). The rabbit polyclonal antibody to transferrin receptor was from Invitrogen, and the HRP-conjugated secondary antibodies were from GE Healthcare.

### Cell Culture, Plasmids and Generation of Stable Cell Lines

H4 human neuroglioma cells were obtained from the American Type Culture Collection (Manassas, VA) and cultured as previously described [19]. The HA- and GFP-tagged APP_695_ construct carrying the substitutions F615P and D664A (APP-F/P-D/A) was generated previously [22]. The GFP-tagged C99 construct was also generated previously [23], and in this study was used as template to introduce the substitutions F38P and D87A (C99-F/P-D/A). In addition, the double-substituted C99 construct F38P, D87A was used as template to introduce substitutions in all five cytosolic lysine residues to arginine (C99-5K/R-F/P-D/A), or in all three cytosolic tyrosine residues to alanine (C99-3Y/A-F/P-D/A). Site-directed mutagenesis was performed using the QuickChange mutagenesis kit (Stratagene, La Jolla, CA). H4 stable cell lines expressing HA-tagged APP-F/P-D/A, C99-F/P-D/A, C99-5K/R-F/P-D/A or C99-3Y/A-F/P-D/A were generated by transfection with Lipofectamine 2000 (Invitrogen) according to the manufacturer’s instructions. Transfected cells were selected in 1 mg/ml of G418 (Invitrogen). Single cell colonies were picked, and those with comparable expression levels were maintained in medium containing 100 µg/ml G418.

### Ubiquitination Assay, Cell Surface Biotinylation, Preparation of Protein Extracts and Immunoblot

For ubiquitination assays, H4 cells stably expressing GFP-tagged C99-F/P-D/A or GFP-tagged C99-5K/R-F/P-D/A were transfected with a construct encoding HA-tagged ubiquitin, using Lipofectamine 2000 (Invitrogen) according to the manufacturer’s instructions. After 16 h cells were washed twice with cold phosphate buffered saline supplemented with 0.1 mM CaCl_2_ and 1 mM MgCl_2_ (PBS-Ca/Mg), and subjected to lysis at 4°C for 1 h with buffer Tx-Bp (50 mM Tris-HCl pH 7.4, 150 mM NaCl, 1 mM EDTA, 1% Triton X-100, and a cocktail of protease inhibitors) supplemented with 10 mM DTT, 5 mM *N*-ethylmaleimide and 10 mM iodoacetamide. C99 was immunoprecipitated from soluble extracts with rabbit anti-GFP serum and protein A-Sepharose, and analyzed by immunoblot with HRP-conjugated anti-HA antibody. Cell surface biotinylation was carried out as previously described [24]. Briefly, cells were washed twice with PBS-Ca/Mg, biotinylated with 1 mM Sulfo-NHS-LC-Biotin (Thermo Scientific) in PBS-Ca/Mg for 30 min, and further incubated with Tris buffered solution (50 mM Tris-HCl pH 7.4) for 10 min to quench free biotin. After washing with PBS-Ca/Mg and subjecting cells to lysis with buffer Tx-Bp, biotinylated proteins were pulled down with Neutravidin Agarose (Thermo Scientific) and analyzed by inmunoblot with rabbit serum to GFP.

### Metabolic Labeling and Immunoprecipitation

Metabolic labeling was carried out as described [25]. Briefly, H4 cells transiently expressing GFP-tagged C99 were pulse-labeled for 15 min with 0.1 mCi/ml of [^35^S]-methionine-cysteine (EasyTag Express Protein Labeling Mix; Perkin Elmer), and chased for 0.5–3 h at 37°C in DMEM supplemented with 10% FBS, 0.06 mg/ml methionine and 0.1 mg/ml cysteine. After chase, cells were washed with cold PBS-Ca/Mg and lysed with buffer Tx-Bp. Aβ was immunoprecipitated from the culture medium with monoclonal 6E10 antibody and protein G-Sepharose, and analyzed by electrophoresis using 10–20% Tricine gels (Invitrogen). C99, the carboxy-terminal fragment-α (C83), and the APP intracellular domain (AICDγ) fragment were immunoprecipitated from soluble extracts with a rabbit anti-GFP serum and protein A-Sepharose, and analyzed by SDS-PAGE as described [19].

### Fluorescence Microscopy

Indirect immunofluorescence staining of fixed, permeabilized cells was carried out as described previously without modifications [26]. Images of fixed cells were acquired with an Olympus FluoView FV1000 scanning unit fitted on an inverted Olympus IX81 microscope, and with a PlanApo 60X oil immersion objective (NA 1.40; Olympus, Melville, NY), using similar settings as described previously [25].

### Densitometric Quantification and Statistical Analysis

The amount of autoradiographic or immunoblot signal was estimated using Image J software version 1.44o (Wayne Rasband, NIH, http://imagej.nih.gov). For each condition, protein bands were quantified from at least three independent experiments. Data analysis was performed using Microsoft Excel for Mac 2011 (Microsoft Corporation). Results are represented in graphs depicting the mean ± standard deviation. Statistical significance was determined by one-tailed *t*-test. *P*-values of *p*<0.05 (*), *p*<0.01 (**), *p*<0.001 (***) were regarded as statistically significant, and are indicated in the figures.

## Results

### Subcellular Localization of C99 in H4 Neuroglioma Cells

**Figure 1:**
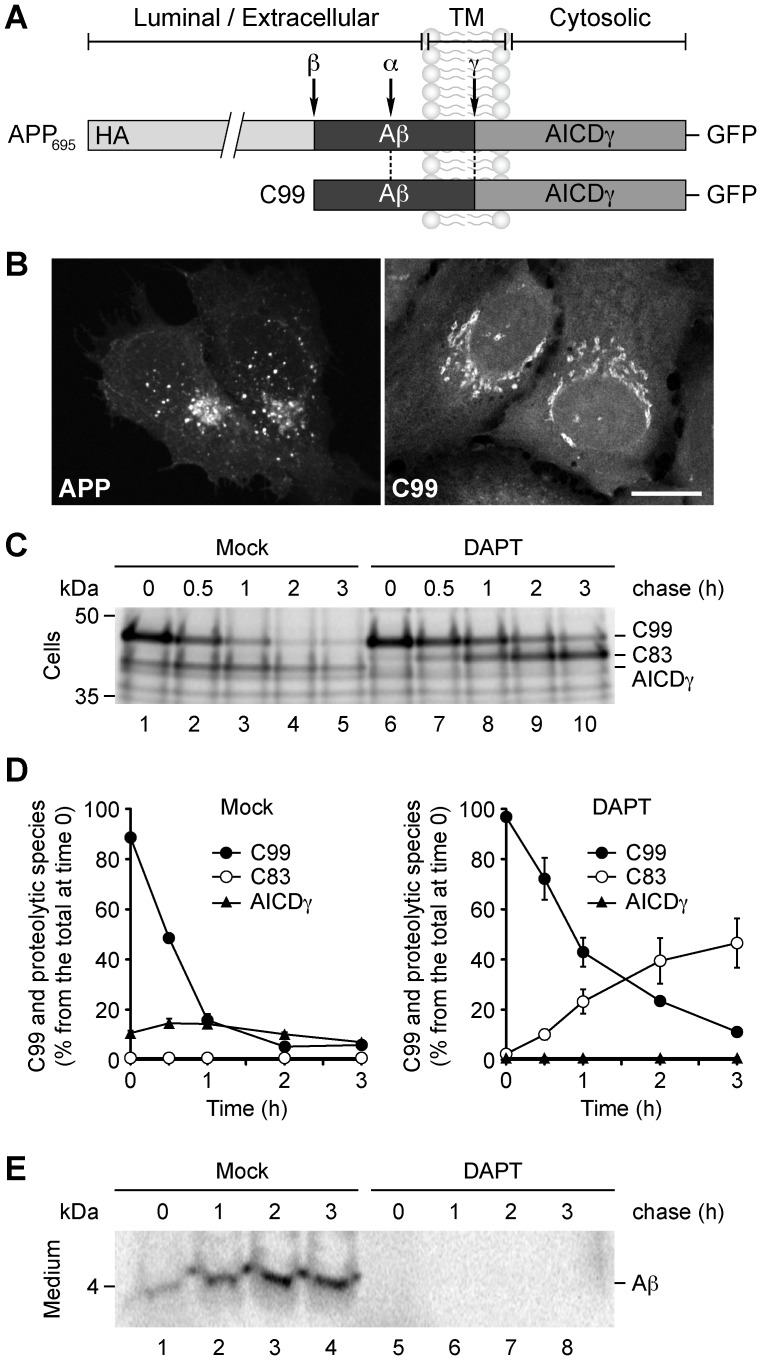
Intracellular localization and proteolytic processing of C99. (A) Schematic representation of GFP-tagged APP and C99 indicating their topological domains and the position of the HA tag, the Aβ peptide, the proteolytic cleavage sites (α, β and γ), and the AICDγ fragment. (B) Fluorescence microscopy analysis of H4 human neuroglioma cells transiently expressing APP-GFP or C99-GFP. Bar, 10 µm. (C–E) H4 cells transiently expressing C99-GFP were left untreated or treated for 16 h with 1 µM DAPT, labeled for 4 hr at 20°C with 1 mCi/ml [^35^S]-methionine-cysteine, and chased at 37°C for the indicated times. C99 and Aβ species were immunoprecipitated from cell lysates with anti-GFP antibody (C), or from the culture medium with 6E10 antibody (E), respectively. Proteins were analyzed on 10%–20% Tricine gels and fluorography. The positions of molecular mass markers are indicated on the left. (D) Densitometric quantification of the levels of C99, C83, and AICDγ shown in C.

To determine in H4 cells the subcellular localization of C99, which is the C-terminal fragment generated after processing of APP by β-secretase, we studied the distribution of a recombinant GFP-tagged C99 (C99-GFP) [19], [23]. Although the addition of a GFP moiety to the rather small C99 (∼10–12 kDa) represents a substantial increment in its size, we chose to use this construct because untagged C99 is difficult to detect in cultured cells due to both its rapid cleavage by γ-secretase and its rapid turnover [6]. Conversely, the addition of a C-terminal GFP tag to C99 partially stabilizes it, allowing further analysis of it [19], [27]. C99-GFP contains the entire amyloidogenic sequence, the transmembrane domain and the cytosolic region of APP, and can be subsequently cleaved by γ-secretase (Fig. 1A). Fluorescence microscopy analysis of H4 cells expressing HA- and GFP-tagged APP (APP-GFP) showed APP highly enriched in endo/lysosomal membranes accompanied by minor Golgi localization (Fig. 1B), as we have previously shown [19]. Nonetheless, this pattern of fluorescence might be the result of a combination of APP-GFP and its proteolytic products. Instead, C99-GFP was localized mainly at the Golgi apparatus (Fig. 1B), as shown by colocalization with endogenous resident proteins (Fig. S1). The localization of C99-GFP seems to be not the result of mistargeting induced by the GFP moiety because HA-tagged C99 showed a similar Golgi localization (Fig. S2, A–C). Moreover, we observed that both an mCherry-tagged C99 and a GFP-tagged C83 also localized at the Golgi apparatus (Fig. S2, D–F). A previous study failed to identify C99-GFP at the Golgi apparatus, presumably due to a higher activity of γ-secretase in the cultured cells used (HEK-293) [27]. Cells expressing either APP-GFP or C99-GFP showed a diffuse cytoplasmic GFP fluorescence, likely corresponding to a cytosolic GFP-tagged AICDγ fragment (AICDγ-GFP). To examine in H4 cells the processing of C99-GFP by γ-secretase, we performed pulse-chase experiments with [^35^S]-methionine-cysteine. Cells were pulsed 15 min, and chased for different periods of time, and C99-GFP and its cleavage products were immunoprecipitated and analyzed by SDS-PAGE and fluorography. We monitored the kinetics of C99-GFP processing without or with N-[N-(3,5-difluorophenacetyl)-L-alanyl]-S-phenylglycine *t*-butyl ester (DAPT), a specific γ-secretase inhibitor. In untreated cells, C99-GFP was rapidly cleaved to AICDγ-GFP, with a half-life of ∼36 min (Fig. 1, C and D, Control). In contrast, in cells treated with DAPT, C99-GFP had a longer half-life of ∼54 min, AICDγ-GFP was almost absent, and there was a steady accumulation of C83-GFP (Fig. 1, C and D, DAPT), which is the product of the non-amyloidogenic proteolytic processing of C99-GFP by α-secretase [18]. As we expected, the addition of DAPT blocked the release of Aβ into the culture medium, as examined with the 6E10 antibody (Fig. 1E). Together, these data demonstrate that in H4 cells, C99-GFP localizes at the Golgi apparatus, is a bona fide γ-secretase substrate, and suggest that the GFP signal at the Golgi in cells expressing APP-GFP might correspond to that of C99-GFP.

### Different Turnover of APP and C99 within Acidic Compartments

**Figure 2:**
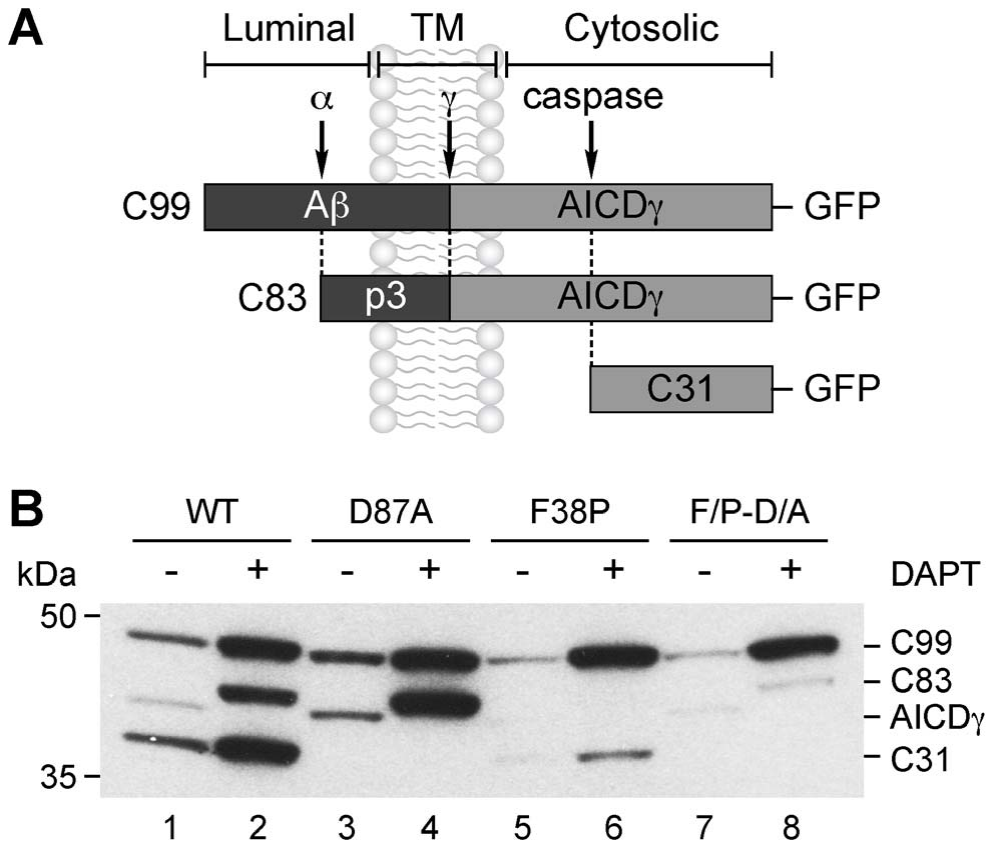
C99 is proteolytically cleaved in different sites. (A) Schematic representation of GFP-tagged C99, C83 and C31 indicating their topological domains, and the position of the Aβ peptide, the p3 peptide, the proteolytic cleavage sites (α, γ and caspase), and the AICDγ fragment. (B) H4 cells transiently expressing wild-type C99-GFP (WT) or C99-GFP with either the D87A mutation, the F38P mutation, or both (F/P-D/A), were left untreated or treated with 1 µM DAPT for 16 h. Cellular extracts were analyzed by immunoblot with anti-GFP antibody. The positions of molecular mass markers are indicated on the left.

**Figure 3:**
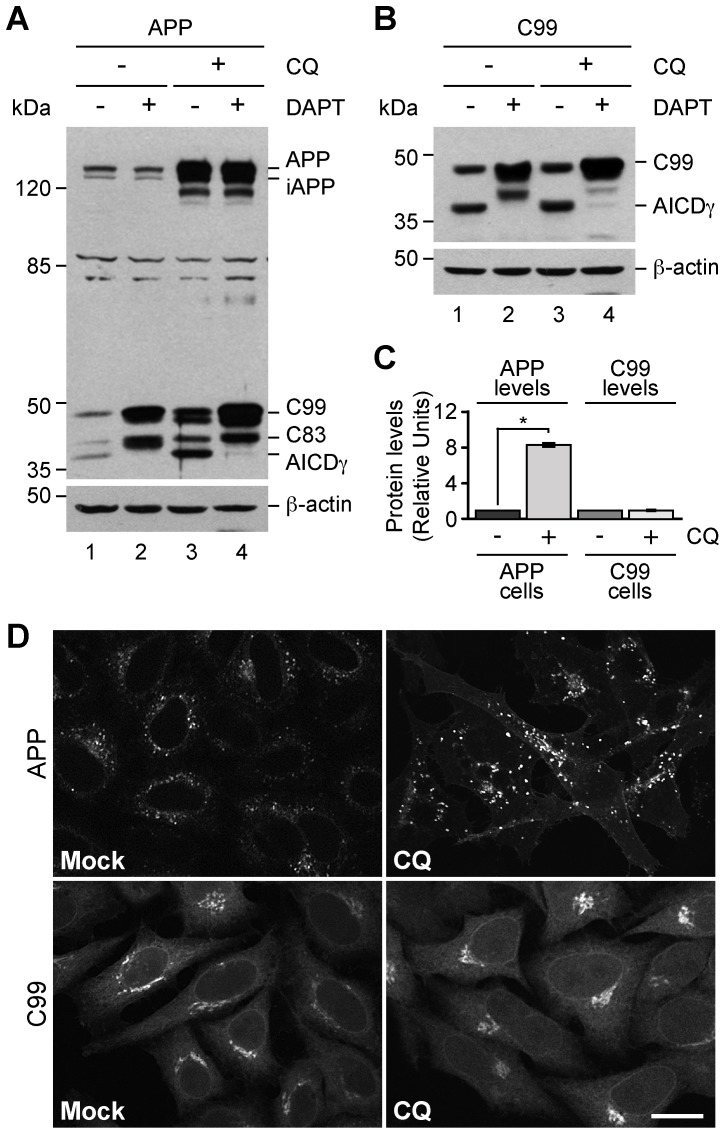
Different response of APP and C99 to CQ. (A–B) H4 cells stably expressing GFP-tagged APP-F/P-D/A (A) or C99-F/P-D/A (B) were left untreated or treated for 16 h either with 1 µM DAPT, 100 µM CQ, or with a combination of 1 µM DAPT and 100 µM CQ. Cellular extracts were analyzed by immunoblot with anti-GFP antibody. The positions of molecular mass markers are indicated on the left. (C) Densitometric quantification of the levels of APP and C99 shown in A and B. Bars represent the mean ± SD (APP n = 7; C99 n = 6). *P<0.05. (D) Confocal fluorescence microscopy of H4 cells stably expressing GFP-tagged APP-F/P-D/A or C99-F/P-D/A left untreated (Control) or treated with 100 µM CQ for 16 h. Bar, 10 µm.

To investigate whether the different localization of APP and C99 is the outcome of differential post-Golgi trafficking, we studied the effect of CQ, a drug that raises the acidic luminal pH of organelles in the late secretory pathway resulting in perturbed endosomal maturation and lysosomal function [28]. To facilitate the analysis, we transiently expressed APP and C99 constructs carrying substitutions that abolish cleavage by α-secretase (F38P), caspases (D87A), or both (F/P-D/A). As we have previously shown for APP [22], these mutations also precluded, in C99, the generation of either C83 (Fig. 2B, lanes 3–4), a caspase-derived C31 fragment (Fig. 2B, lanes 5–6) [19], or both fragments (Fig. 2B, lanes 7–8). Additionally, we found that the double mutant C99-F/P-D/A (referred to as C99) is a good substrate for γ-secretase, giving rise to AICDγ. In the presence of DAPT, AICDγ was absent, while C99 was present at expected levels (Fig. 2B, lanes 7–8). We then compared in H4 cells stably expressing APP or C99 the effect of CQ in the absence or presence of DAPT. In cells expressing APP, we found that in response to CQ, there was an ∼8-fold increase in the levels of the mature and immature forms of APP, but with a corresponding increase of only a ∼2.5-fold in the levels of C99 and AICDγ (Fig. 3, A and C). A similar accumulation upon CQ treatment was observed with untagged, wild-type APP (i.e., APP without F/P-D/A mutations) (Fig. S3, lanes 2 and 5). In contrast, in cells expressing C99 incubated with CQ, the levels of C99 were mostly unaffected (Fig. 3, B and C). Similarly, no accumulation was observed in cells expressing HA-tagged, wild-type C99 (i.e., C99 without F/P-D/A mutations) (Fig. S4A, lanes 1 and 2). Interestingly, no accumulation of C83 was observed in cells expressing HA-tagged, wild-type C83 (i.e., C83 without F/P-D/A mutations) (Fig. S4B, lanes 1 and 2). These results suggest that in cells expressing APP, the increase in C99 and AICDγ levels upon CQ treatment could be due to higher levels of APP in an acidic compartment, and subsequently increased cleavage by both β-secretase and γ-secretase, rather than to an inhibition of C99 proteolytic processing and/or lysosomal turnover. Although these experiments cannot rule out these latter possibilities, both the disproportionate increase in the levels of APP and C99, and the increase in AICDγ levels suggest at least that APP is a better substrate for lysosomal degradation than C99, and that a different mechanism should account for its disproportionate accumulation. In line with this interpretation, immunofluorescence microscopy showed that in response to CQ, APP accumulates in endosomes, as judged by colocalization with endosomal markers (data not shown), but not in lysosomes (data not shown), while C99 localization at the Golgi seemed unchanged (Fig. 3D). Similar results were obtained with bafilomycin A1, a specific inhibitor of the vacuolar H^+^-ATPase (data not shown). Altogether, our findings indicate that APP, but not C99, is mostly degraded within acidic compartments, strongly suggesting that the post-Golgi trafficking of APP and C99 are different.

### Turnover of C99 within the Early Secretory Pathway by the Proteasome

**Figure 4:**
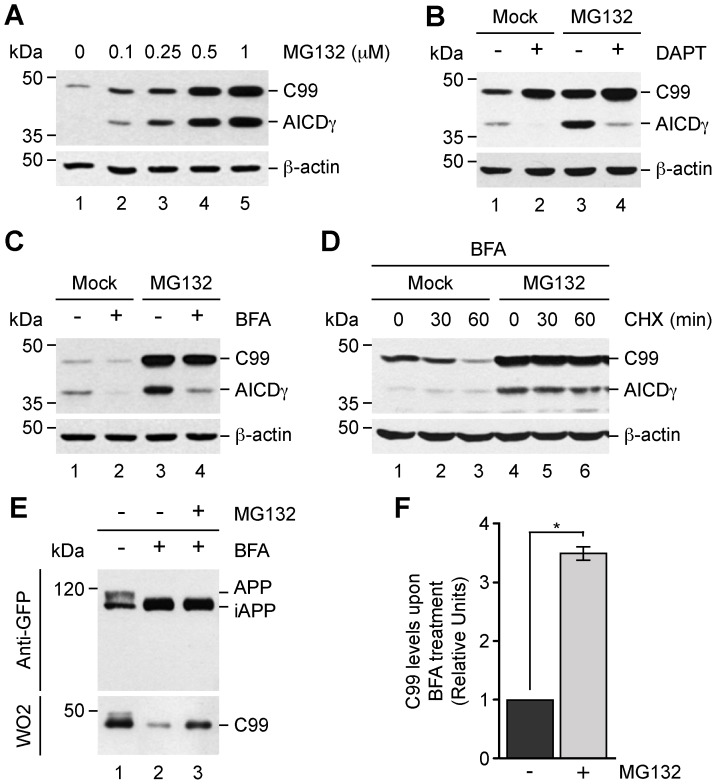
C99 is degraded after redistribution to the endoplasmic reticulum. H4 cells stably expressing GFP-tagged C99-F/P-D/A were treated as follows: (A) with increasing concentrations of MG132 for 4 h; (B) left untreated or treated either with 1 µM DAPT for 16 h, 1 µM MG132 for 4 h, or 1 µM DAPT for 12 h followed by a combination of 1 µM DAPT and 1 µM MG132 for 4 h; (C) left untreated or treated for 4 h either with 5 µg/ml BFA, 1 µM MG132, or a combination of 1 µM MG132 and 5 µg/ml BFA; or (D) pretreated with 5 µg/ml BFA without or with 1 µM MG132 for 4 h followed by CHX-chase for 0–60 min without or with 1 µM MG132. (E) H4 cells stably expressing GFP-tagged APP-F/P-D/A were left untreated or treated for 4 h either with 5 µg/ml BFA, or a combination of 5 µg/ml BFA and 1 µM MG132. Cellular extracts were analyzed by immunoblot with anti-GFP antibody (A–E), or WO2 monoclonal antibody to detect C99 in cells expressing GFP-tagged APP-F/P-D/A (E). Immunoblot with anti-β-actin antibody was used as loading control. The positions of molecular mass markers are indicated on the left. (F) Densitometric quantification of the levels of C99 shown in E. Bars represent the mean ± SD (n = 4). *P<0.05.

Because we observed no degradation of C99 within acidic compartments, we decided to study whether C99 turnover was dependent on proteasomal degradation. To test this possibility, H4 cells stably expressing C99 were incubated with different concentrations of the proteasome inhibitor MG132. Surprisingly, we found that C99 and AICDγ accumulate upon treatment with MG132 in a dose-dependent manner (Fig. 4A). We obtained a similar result with lactacystin, another proteasome inhibitor (data not shown). Pretreatment of cells with DAPT resulted in further accumulation of C99, and confirmed the identity of AICDγ in response to MG132 (Fig. 4B). Together, these data strongly indicated that C99 is a substrate for proteasomal degradation, and prompted us to investigate whether the accumulation of C99 with MG132 was connected to ERAD. First, we tested the effect on C99 of the treatment with BFA, a drug that causes redistribution of Golgi-localized proteins back to the ER [29]. In cells treated with BFA there was a reduction in C99 and AICDγ levels (Fig. 4C), suggesting that delivery of C99 back to the ER might enhance its degradation by ERAD with a consequent reduction in its proteolytic processing by γ-secretase. In line with this interpretation, when cells were treated with MG132 in the presence of BFA, C99 was again accumulated (Fig. 4C). On the other hand, the lower levels of AICDγ in cells treated with BFA could also be explained by a reduction in C99 processing by γ-secretase within the ER. In effect, the levels of AICDγ were highly reduced when cells were treated with MG132 and BFA compared to cells treated with MG132 only (Fig. 4C). These results were corroborated by cycloheximide-chase experiments, in which the turnover of C99 was greatly delayed in cells treated with BFA during proteasome inhibition by MG132 (Fig. 4D). Finally, fluorescence microscopy analyses confirmed the redistribution of C99 to the ER in cells treated with MG132 and BFA (Fig. S5). Similar results were observed with HA-tagged C99 (data not shown). Because the proteolytic processing of APP by β-secretase within the ER has been proposed as a very unlikely event [30], we evaluated whether C99 generated from APP is degraded by ERAD. We used H4 cells stably expressing APP-F/P-D/A, a validated model to study the generation of C99 by endogenous β-secretase [22]. With this experimental setup, treatment with BFA results in the accumulation of immature APP, leading to a significant reduction in C99 levels [22]. However, when cells were treated with BFA in combination with MG132, the generation of C99 from APP was restored to levels close to those of untreated cells (Fig. 4E), with a ∼3.5-fold increase in C99 levels compared to those in cells treated with BFA alone (Fig. 4F). Altogether, these results demonstrate that C99 can be produced at the ER and subsequently efficiently degraded by the proteasome, resulting in the reduction of C99 proteolytic processing by γ-secretase.

### Degradation of C99 at the ER is Dependent on the Ubiquitination of its Cytosolic Lysine Residues

**Figure 5:**
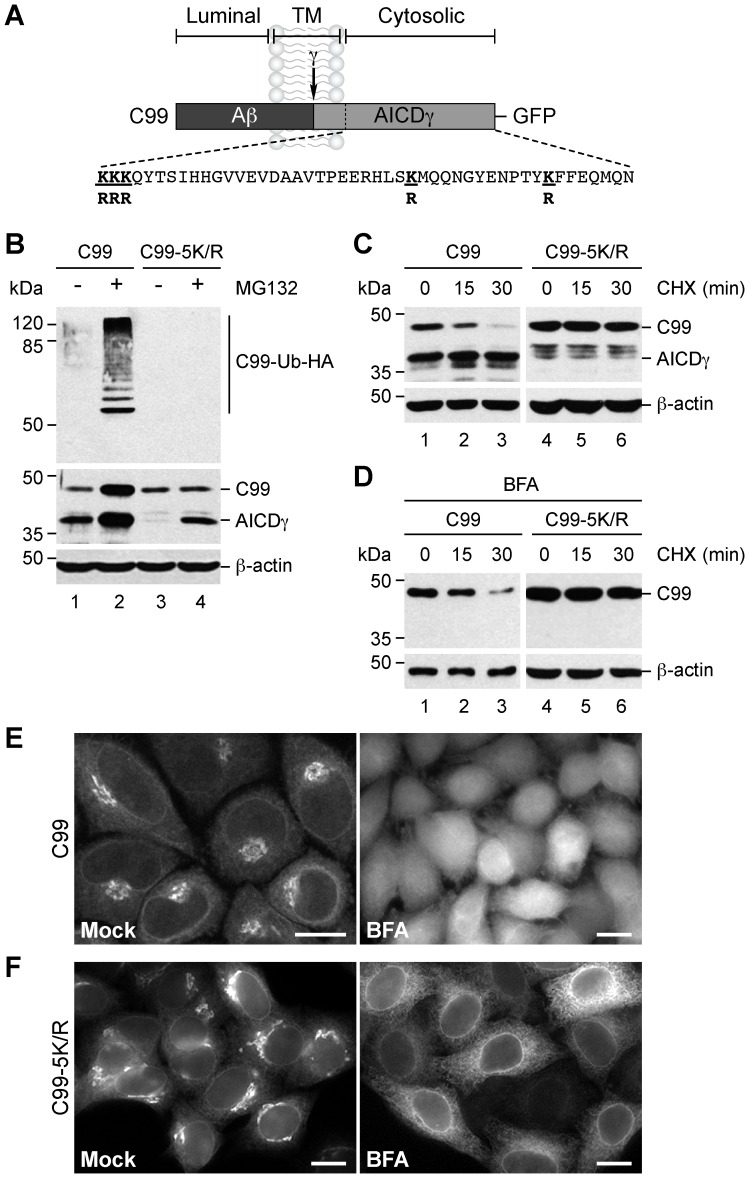
Degradation of C99 after redistribution to the endoplasmic reticulum requires polyubiquitination of its cytosolic lysine residues. (A) Schematic representation of GFP-tagged C99 indicating its topological domains, the position of the Aβ peptide, the γ-secretase cleavage site, the AICDγ fragment, and the sequence of the cytosolic tail highlighting the substitutions in its five lysine residues (bold underline). (B–D) H4 cells stably expressing GFP-tagged C99-F/P-D/A (C99) or C99-5K/R-F/P-D/A (C99-5K/R) were processed as follows: (B) transfected with HA-tagged ubiquitin and left untreated or treated with 1 µM MG132 for 4 h, and after denaturation soluble extracts immunoprecipitated with anti-GFP antibody; (C) incubated with 150 µg/ml CHX and 40 µg/ml chloramphenicol for 0–30 min; or (D) pretreated with 5 µg/ml BFA for 1 h before further incubation with BFA and the combination of CHX and chloramphenicol for 0–30 min. Proteins were analyzed by immunoblot with HRP-conjugated anti-HA antibody (B; C99-Ub-HA), or anti-GFP antibody (B–D). Immunoblot with anti-β-actin antibody was used as loading control. The positions of molecular mass markers are indicated on the left. (E–F) Confocal fluorescence microscopy of cells stably expressing GFP-tagged C99 (E) or C99-5K/R (F) left untreated (Control) or treated with 5 µg/ml BFA for 1 h. Bars, 10 µm.

Because the degradation of membrane proteins by ERAD is usually dependent on ubiquitination, we determined whether ubiquitin could play a role in the turnover of C99 at the ER. It has been shown that APP can be ubiquitinated *in vitro* [31] and *in vivo* [32], and like APP, C99 contains five lysine residues on its cytosolic region (Fig. 5A). To study ubiquitination, we generated a version of C99 in which we substituted arginine residues for all of its cytosolic lysine residues, a construct that we referred in this study to as C99-5K/R. H4 cells stably expressing C99 or C99-5K/R were transiently transfected with HA-tagged ubiquitin, and C99 species were immunoprecipitated and analyzed by immunoblot. Strikingly, we observed that C99 was polyubiquitinated, a modification that was detected only when degradation was prevented by MG132 (Fig. 5B, lanes 1–2), and that was completely abolished in the absence of lysine residues in the cytosolic domain of C99 (Fig. 5B, lanes 3–4). No accumulation of C99-5K/R was observed in response to MG132 (Fig. 5B, lane 4), indicating that ubiquitination is necessary for proteasomal degradation of C99. Interestingly, MG132 increased the levels of AICDγ even in the absence of ubiquitination (Fig. 5B, lanes 2 and 4), supporting the notion that processing by γ-secretase is favored during proteasome inhibition. Cycloheximide chase-experiments showed that the absence of cytosolic lysine residues rendered C99 more stable both in the absence (Fig. 5C) and presence of BFA (Fig. 5D), recapitulating the effect of MG132 on ubiquitinated C99 (Fig. 4D). In addition, we observed moderate but reproducible higher levels of C99-5K/R in untreated cells (Fig. 5, B–D) that correlated with enhanced Golgi fluorescence (Figs. 5F and S6), consistent with less proteasomal degradation. In contrast, in cells treated with BFA, C99-5K/R accumulated in the ER with little or no cytosolic fluorescence (Fig. 5F). Altogether, our findings demonstrate that ubiquitination of cytosolic lysine residues is a necessary modification for the degradation of C99 in the ER.

### Proteasome Inhibition Triggers Degradation of C99 within Lysosomes

**Figure 6:**
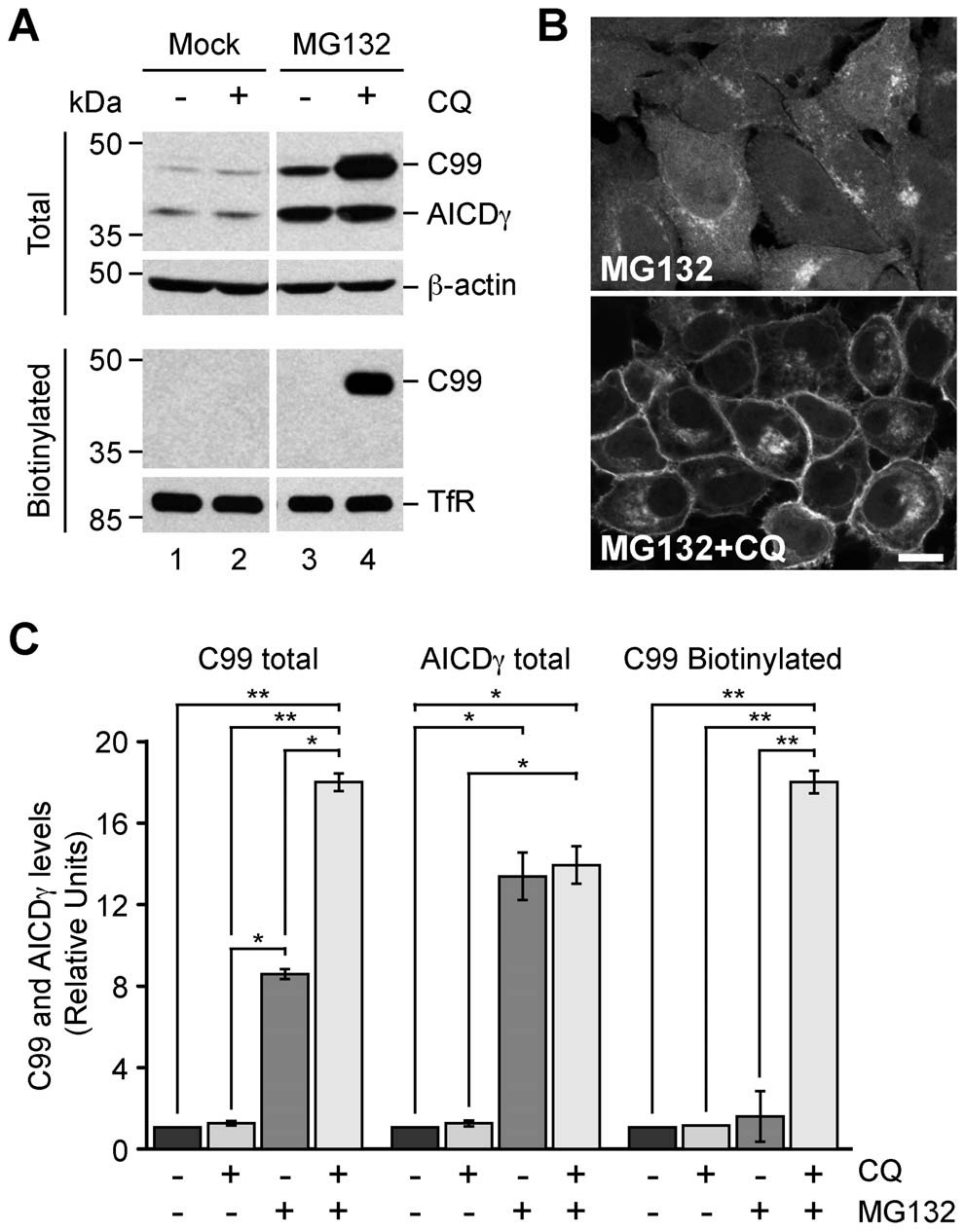
Accumulation of C99 at the cell surface in response to MG132 and CQ. (A) H4 cells stably expressing GFP-tagged C99-F/P-D/A were left untreated or treated for 16 h either with 100 µM CQ, 1 µM MG132, or with a combination of 100 µM CQ and 1 µM MG132. Cells were biotinylated on the cell surface with Sulfo-NHS-LC-Biotin and soluble extracts pulled down with NeutrAvidin-agarose. Total and biotinylated proteins were analyzed by immunoblot with anti-GFP antibody. Immunoblot with anti-β-actin or anti-transferrin receptor (TfR) antibodies was used as loading control for total or biotinylated proteins, respectively. The positions of molecular mass markers are indicated on the left. (B) Confocal fluorescence microscopy of H4 cells stably expressing GFP-tagged C99-F/P-D/A treated for 16 h either with 1 µM MG132 or with a combination of 1 µM MG132 and 100 µM CQ. Bar, 10 µm. (C) Densitometric quantification of the levels of C99 and AICDγ shown in A. Bars represent the mean ± SD (n = 4). *P<0.05; **P<0.01.

Proteasome inhibition causes accumulation of several membrane proteins at the ER due to a disruption of the ERAD pathway [33]. We investigated the fate of C99 in this condition and hypothesized that it could be further targeted to lysosomes for degradation. To test this scenario, we disrupted the lysosomal function with CQ during proteasome inhibition by MG132. As shown before, in H4 cells stably expressing C99, CQ alone did not cause any changes in C99 levels (Fig. 6A, lane 2). In contrast, CQ caused a significant ∼18-fold increase in the levels of C99 when proteasomal degradation was inhibited (Fig. 6, A and C), indicating that C99 is indeed targeted to lysosomes, and suggesting that the degradation of certain cargos within lysosomes might be coupled to ERAD status. A similar response was observed for HA-tagged, wild-type C99 (Fig. S4A) or HA-tagged, wild-type C83 (Fig. S4B), suggesting a common turnover mechanism for these C-terminal fragments. Surprisingly, this condition caused a strong accumulation of C99 at the plasma membrane, as shown by cell surface biotinylation assays (Fig. 6A, lane 4), and fluorescence microscopy analysis (Fig. 6B). This effect on C99 seemed specific to C99 because the same conditions resulted in no change in the cell surface levels of the transferrin receptor (Fig. 6A), a protein that constitutively undergoes endocytosis. As it has been reported for other endocytic processes [28], [34], accumulation of C99 at the cell surface presumably is the consequence of CQ also disrupting C99 internalization and delivery to lysosomes. Altogether, our results indicate that proteasome inhibition elicited the trafficking of C99 from the ER to lysosomes, highlighting a putative crosstalk between these degradative compartments.

### Targeting of C99 to Lysosomes during Proteasome Inhibition is Independent of its Tyrosine Residues

**Figure 7:**
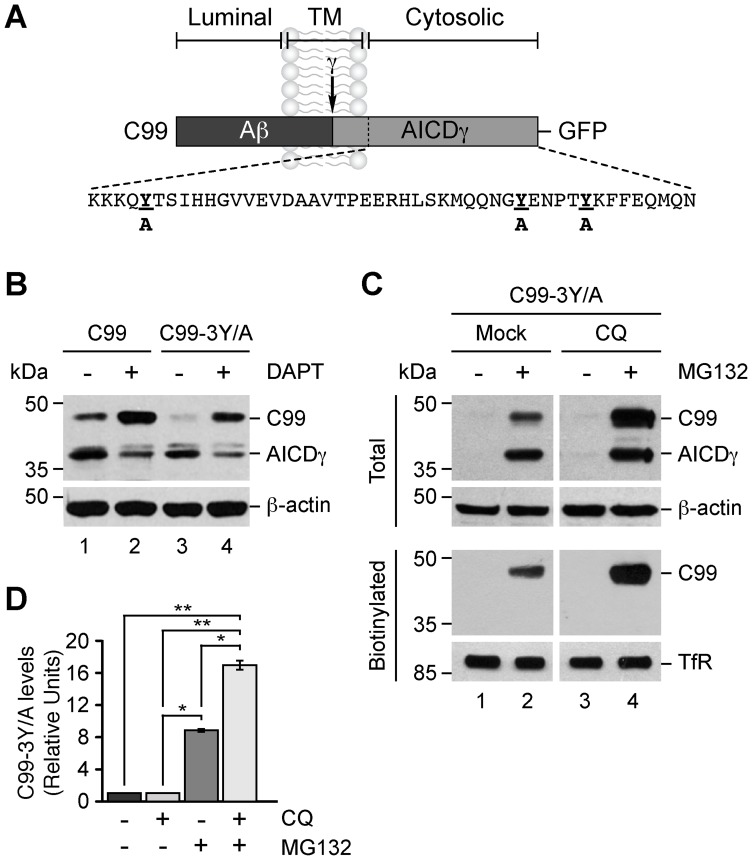
Accumulation of C99 in response to MG132, CQ and lack of its cytosolic tyrosine residues. (A) Schematic representation of GFP-tagged C99 indicating its topological domains, the position of the Aβ peptide, the γ-secretase cleavage site, the AICDγ fragment, and the sequence of the cytosolic tail highlighting the substitutions in its three tyrosine residues (bold underline). (B) Immunoblot analysis of H4 cells stably expressing GFP-tagged C99-F/P-D/A (C99) or C99-3Y/A-F/P-D/A (C99-3Y/A). Cells were left untreated or treated with 1 µM DAPT for 16 h and subsequently analyzed by immunoblot with anti-GFP antibody. Immunoblot with anti-β-actin was used as loading control. The positions of molecular mass markers are indicated on the left. (C) H4 cells stably expressing C99-3Y/A were left untreated or treated for 16 h either with 1 µM MG132, 100 µM CQ, or with a combination of 1 µM MG132 and 100 µM CQ. Cells were biotinylated on the cell surface with Sulfo-NHS-LC-Biotin and soluble extracts pulled down with NeutrAvidin-agarose. Total and biotinylated proteins were analyzed by immunoblot with anti-GFP antibody. Immunoblot with anti-β-actin or anti-transferrin receptor (TfR) antibodies was used as loading control for total or biotinylated proteins, respectively. The positions of molecular mass markers are indicated on the left. (D) Densitometric quantification of C99-3Y/A left untreated or treated for 16 h either with 100 µM CQ, 1 µM MG132, or with a combination of 100 µM CQ and 1 µM MG132. Bars represent the mean ± SD (n = 3). *P<0.05; **P<0.01.

To determine whether the turnover of C99 was dependent on its cytosolic tyrosine residues, we generated the construct C99-3Y/A-F/P-D/A in which we substituted three alanine residues for three tyrosine residues that C99 contains in its cytosolic tail, a construct that we referred as C99-3Y/A (Fig. 7A). Previous studies have demonstrated that these tyrosine residues play a role in the internalization of APP [35], [36], but not on its delivery to the cell surface [22], however it is unclear the role of these residues in C99. We observed that like C99, C99-3Y/A was processed to AICDγ (Fig. 7B, lanes 1 and 3), and that treatment with DAPT also precluded AICDγ formation (Fig. 7B, lanes 2 and 4), demonstrating that processing of C99 by γ-secretase occurs independently of all cytosolic tyrosine residues. Likewise, inhibition of proteasomal degradation by MG132 resulted in accumulation of C99-3Y/A (Fig. 7C, lanes 1 and 2), demonstrating that turnover of C99 by the proteosome is also independent of all cytosolic tyrosine residues (Fig. 7C). Similar to the effect on C99 or on HA-tagged, wild-type C99 and on HA-tagged, wild-type C83 (Figs. 6A and S4), CQ did not cause apparent changes in the levels of C99-3Y/A (Fig. 7C, lanes 1 and 3). In contrast, CQ caused a significant ∼17-fold increase in the levels of C99-3Y/A when proteasomal degradation was inhibited by MG132 (Fig. 7C, lanes 1 and 4), indicating that C99 can be targeted to an acidic compartment upon proteosomal inhibition independent of all cytosolic tyrosine residues. On the other hand, biotinylation assays showed that the levels of C99-3Y/A at the cell surface were undetectable (Fig. 7C), confirming that the majority of C99 is not trafficking constitutively to the plasma membrane. In contrast, we found that C99-3Y/A strongly accumulates at the cell surface when both the proteasome is inhibited by MG132 and the lysosomal function is disrupted with CQ (Fig. 7C, lane 4). Similar results were observed by fluorescence microscopy analysis (data not shown). Interestingly, in cells treated with only MG132 we observed accumulation of C99-3Y/A at the cell surface, albeit to a lesser extent than in cells treated with MG132 and CQ (Fig. 7C, lanes 2 and 4). This suggests that a fraction of C99 is diverted to the cell surface when the proteasome is inhibited, and that for further endocytosis one or more of its cytosolic tyrosine residues is necessary. Together, these findings indicate that proteasome inhibition targets C99 to lysosomes through a pathway that is independent of all cytosolic tyrosine residues.

### Ubiquitin-independent Degradation of C99 within Lysosomes

**Figure 8:**
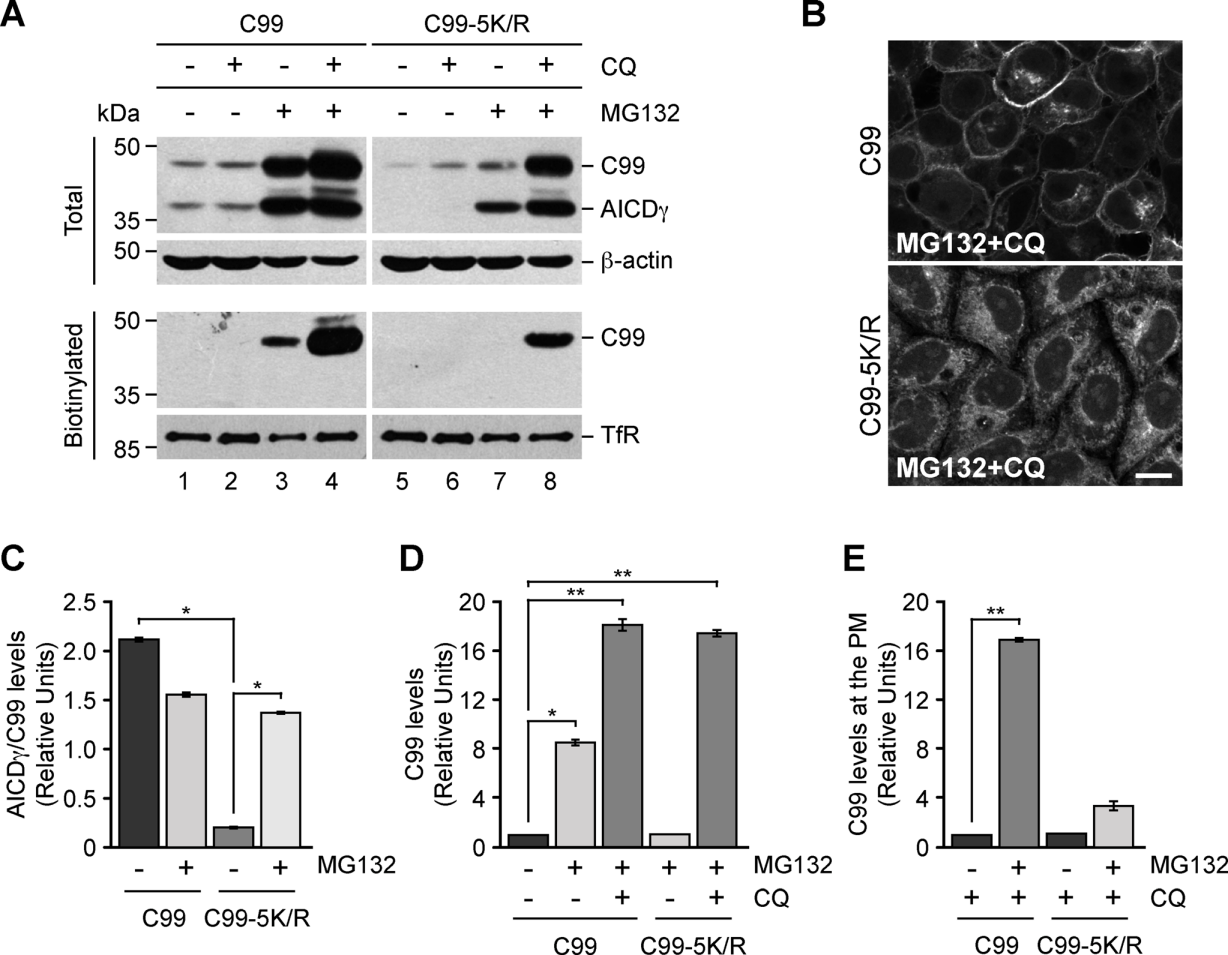
Degradation of C99 in acidic compartments is independent of its ubiquitination. (A) H4 cells stably expressing GFP-tagged C99-F/P-D/A (C99) or C99-5K/R-F/P-D/A (C99-5K/R) were left untreated or treated for 16 h either with 100 µM CQ, 1 µM MG132, or with a combination of 100 µM CQ and 1 µM MG132. After cells were biotinylated on the cell surface with Sulfo-NHS-LC-Biotin and soluble extracts pulled down with NeutrAvidin-agarose, total and biotinylated proteins were analyzed by immunoblot with anti-GFP antibody. Immunoblot with anti-β-actin or anti-transferrin receptor (TfR) antibodies was used as loading control for total or biotinylated proteins, respectively. The positions of molecular mass markers are indicated on the left. (B) Confocal fluorescence microscopy of H4 cells stably expressing GFP-tagged C99-F/P-D/A (C99) or C99-5K/R-F/P-D/A (C99-5K/R) treated with a combination of 1 µM MG132 and 100 µM CQ for 16 h. Bar, 10 µm. (C) Densitometric quantification of the ratio AICDγ/C99 of H4 cells stably expressing GFP-tagged C99-F/P-D/A (C99) or C99-5K/R-F/P-D/A (C99-5K/R) left untreated or treated for 16 h with 1 µM MG132. (D) Densitometric quantification of the levels of C99 in H4 cells stably expressing GFP-tagged C99-F/P-D/A (C99) or C99-5K/R-F/P-D/A (C99-5K/R) left untreated or treated for 16 h with 1 µM MG132, or with a combination of 100 µM CQ and 1 µM MG132. (E) Densitometric quantification of the levels of C99 at the plasma membrane (PM) in H4 cells stably expressing GFP-tagged C99-F/P-D/A (C99) or C99-5K/R-F/P-D/A (C99-5K/R) left untreated or treated for 16 h with 1 µM MG132, or with a combination of 100 µM CQ and 1 µM MG132. Bar represents the mean ± SD (n = 3). *P<0.05; **P<0.01.

Because delivery of transmembrane proteins to lysosomes for degradation is often dependent on the ubiquitination of their cytosolic domains, we investigated the role of ubiquitination in the delivery of C99 to lysosomes during proteasome inhibition. To this end, H4 cells stably expressing the mutant C99-5K/R that lacks all putative ubiquitination sites were treated with CQ during proteasome inhibition by MG132, and the levels of C99 were compared to those in cells expressing C99. Immunoblot analysis showed that in untreated cells, the ratio of the levels of AICDγ over C99-5K/R were reduced to ∼9% of the ratio of AICDγ over C99 (Fig. 8, A, lanes 1 and 5, and C). However, upon proteosome inhibition by MG132, the ratio of AICDγ over C99-5K/R and that of AICDγ over C99 were very similar (Fig. 8, A, lanes 4 and 8, and C), demonstrating that efficient γ-secretase processing of C99 is dependent on proteasomal activity, but independent of its cytosolic, ubiquitinable lysine residues. Moreover, we observed a similar ∼18-fold increase in the levels of either C99 or C99-5K/R in cells treated with CQ and MG132 (Fig. 8A, lanes 4 and 8; and Fig. 8D), suggesting that C99 can be degraded within lysosomes even in the absence of ubiquitination. Unexpectedly, the inhibition of protein degradation by treatment with CQ and MG132 resulted in a reduction of the levels of C99-5K/R at the cell surface to a ∼7% of the levels of C99, as observed by both biotinylation (Fig. 8A, lanes 4 and 8, and E) and fluorescence microscopy analysis (Fig. 8B), indicating that ubiquitination might also play a role in the trafficking of C99 to the cell surface.

## Discussion

**Figure 9:**
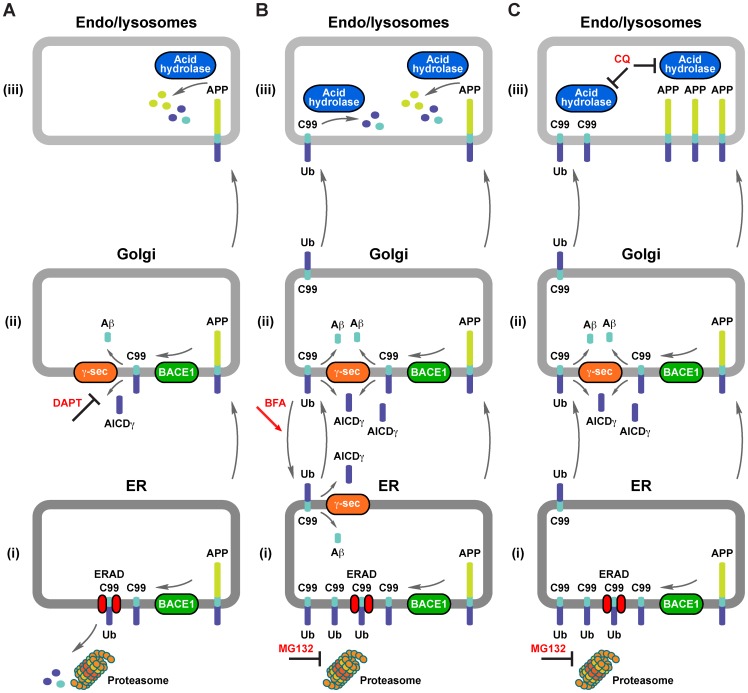
Proposed processing and turnover routes of C99. (A) (*i*) A small fraction of newly-synthesized APP in the endoplasmic reticulum (ER) can be a substrate of BACE1 that generates C99. Ubiquitinated (Ub) C99 can be a substrate of the endoplasmic reticulum-associated protein degradation (ERAD) pathway to ultimately be degraded by the proteasome. (*ii*) En route through the secretory pathway, a fraction of APP at the Golgi apparatus can also be a substrate of BACE1 that generates C99, which subsequently can be a substrate of γ-secretase (γ-sec) activity that generates Aβ peptides and cytosolic AICDγ, a proteolytic processing that can be inhibited by DAPT. (*iii*) Finally, within endo/lysosomal compartments APP can be degraded by acid hydrolases. (B) (*i*) Upon MG132 inhibition, ubiquitinated C99 accumulates within the ER. Ubiquitinated C99 can exit the ER and reach the Golgi apparatus. (*ii*) Both ubiquitinated C99 and C99 generated from APP can be cleaved at the Golgi apparatus by γ-secretase activity. Upon Brefeldin A (BFA) treatment, C99 can be relocated from the Golgi apparatus to the ER where it can be also cleaved by γ-secretase activity. (*iii*) Both APP and the excess of C99 can be degraded by acid hydrolases. (C) (*i*) Upon MG132 treatment, and (*ii*) the generation of an excess of C99 at the Golgi apparatus, (*iii*) chloroquine (CQ) treatment results in accumulation of both APP and C99 within endo/lysosomal compartments. For simplicity, other APP metabolites, such as sAPPβ, which is the other product of BACE1 activity on APP, or the C31 fragment, are not depicted.

Substantial evidence indicates that the level of C99 is a key determinant of Aβ generation in AD [37]. Thus, it is reasonable to speculate that cells must deploy multiple mechanisms to ensure that C99 is rapidly destroyed as soon as it is generated. The best-known non-amiloydogenic, seemingly physiologic proteolytic processing of C99 is by α-secretase [18]. However, it is plausible that organelles with robust membrane protein degradative functions, such as the ER and lysosomes, can also play a role in C99 disposal. Although there are a number of studies demonstrating the production of Aβ_42_ within the ER [12], [13], [14], generation of C99 within this compartment is still a matter of controversy. This is mainly because several studies have shown C99 generation at different intracellular sites [19], [36], [38], [39], [40], [41], [42], and other studies have reported different subcellular distribution of the secretases [2], [5], [17], [22], [42]. The reason for this debate lies in part in findings showing that BFA greatly reduces the levels of C99 and Aβ, leading to the notion that APP is not cleaved by β-secretase within the ER [30], [43]. Contrary to this interpretation, the results shown in this report indicate that the decrease in C99 levels produced by BFA could be the consequence of efficient ubiquitin-dependent proteasomal degradation of C99 after redistribution to the ER, strongly implicating the contribution of the ERAD pathway in this process (Figure 9, A and B). Several independent lines of evidence support this conclusion. For instance, it has been shown that *in vitro* translation of APP also produces C99 [44], that C99 and Aβ_42_ are substrates for proteasomal degradation [6], [45], [46], and that the knockdown of the ubiquitin ligase HRD1, a component of the ERAD pathway, can cause accumulation of APP and an increase in Aβ levels [47]. Because ERAD participates in quality control by eliminating missfolded proteins, it seems likely that degradation of C99 through this pathway might occur in response to failures in its folding, preventing Aβ generation. In fact, disruption of the ER quality control machinery leads to an increase in the levels of Aβ_42_ *in vivo* [48]. In this context, it has been shown that ubiquitination of immature APP is regulated by ubiquilin-1, an ER quality control chaperone linked to late-onset AD [32]; hence, it would be interesting to explore its role in C99 turnover at the ER.

While many reports have shown that APP is localized mainly in endo/lysosomal compartments [19], [20], [21], it is also well accepted that APP attains a steady-state distribution that includes the Golgi apparatus and the plasma membrane [38], [49]. The proportion of APP in each of these compartments, and the fate of each of these pools in terms of processing and turnover, however, is currently unknown. CTFs, on the other hand, can interact with the γ-secretase complex, an interaction favored at the Golgi apparatus [17], [42]. Therefore, both the localization of C99 at the Golgi apparatus and its redistribution to the ER upon BFA treatment are consistent with these previous reports. So far, there have been no systematic studies showing whether the CTFs are distributed differently than APP, and whether the interaction of CTFs with γ-secretase could explain their localization at the Golgi apparatus. Because the different secretases that participate in the proteolytic processing of APP might have different distributions within the cell, a certain proportion of the CTFs could also be generated at these different sites. Likewise, even though at any given time, APP and the CTFs can be in the same compartment, they could establish different molecular interactions, and consequently the protein sorting machinery could also be recognizing them differently. A reasonable possibility is that even minor BACE1 activity on APP could generate CTFs at the ER and the Golgi apparatus, and that these CTFs could be less well recognized by the sorting machinery, giving rise to a disproportionate distribution.

In addition to the above postulated role of ERAD, we propose that the Golgi apparatus must act as an additional checkpoint for the degradation of C99, and at two levels. First, by rerouting C99 back to the ER for ERAD removal, using a route that is highly efficient for the normal transport of both endogenous (KDEL-R; [50]) and exogenous (STxB; [51]) proteins (Figure 9B). Alternatively, the Golgi apparatus could sense ERAD impairment or ER overload, subsequently rerouting C99 to lysosomes for degradation to reduce Aβ production in the Golgi (Figure 9B) [19], [52]. A similar mechanism has been postulated for ERAD substrates in both yeast [53] and mammalian cells [54], highlighting a putative crosstalk between the ER and lysosomal degradation to avoid protein accumulation and toxicity. Our results imply that C99 can be degraded in lysosomes as an alternative to ERAD when the proteasomal activity is impaired (Figure 9, B and C). Such degradation relied in part on C99 tyrosine-dependent internalization [36], but is independent of its ubiquitination. A similar ubiquitin-independent endocytic sorting for degradation in lysosomes has been reported for PAR1, a G-protein coupled receptor [55]. Thus, ubiquitination is key for the degradation of C99 at the ER but not in lysosomes. Further studies are necessary to decipher the requirements for C99 incorporation into lysosomes during ERAD impairment. Consequently, our data suggest the possibility that C99 generated in endo/lysosomal compartments is subjected to a turnover mechanistically different from that of the turnover of C99 generated in the Golgi apparatus, which could explain how amyloid peptides of different length are formed [56]. Overall, our results show that APP, C99 and C83 can be proteolytically processed at different subcellular locations and under different physiological conditions. In fact, our studies show for the first time that the levels of CTFs generated in early compartments of the secretory pathway could be affected by ERAD, as it has also been shown seems to be the case for APP [57]. It would be important to know now what physiological conditions favor these different turnover pathways in terms of Aβ generation.

Although recent reports have shown that APP is a substrate for ubiquitination, presumably for degradation in lysosomes [31], [32], it is unclear whether ubiquitination has additional roles on APP. Unexpectedly, we found that ubiquitination is important for the translocation of C99 to the cell surface. This suggests that this post-translational modification could serve as a signal for the trafficking of C99, similar to the role that ubiquitination has on the traslocation of GLUT4 from intracellular insulin-sensitive stores to the cell surface [57]. In agreement with this possibility, increased ubiquitination of APP by overexpression of FBL2, a component of the E3 ubiquitin ligase complex, results in increased localization of APP at the cell surface [31]. Our findings therefore imply that ubiquitination plays unexpected roles in C99 turnover, processing, and trafficking that need further investigation.

## Supporting Information

Figure S1 **Intracellular localization of C99-GFP.** H4 cells transiently expressing GFP-tagged C99 grown on coverslips were fixed, permeabilized, and double-labeled with mouse monoclonal antibodies to EEA1 or CD63, and sheep antibody to TGN46, followed by Alexa-594-conjugated donkey anti-mouse IgG (red channel), and Alexa-647-conjugated donkey anti-sheep IgG (blue channel). Stained cells were examined by confocal fluorescence microscopy. Merging of the images in the green, red, and blue channels generated the fourth picture in the first and second row; yellow indicates overlapping localization of the green and red channels, cyan indicates overlapping localization of the green and blue channels, magenta indicates overlapping localization of the red and blue channels, and white indicates overlapping localization of the red, green, and blue channels. Bar, 10 µm.
(TIF)

Figure S2 **Intracellular localization of epitope-tagged C99 and C83.** H4 cells grown on coverslips were transiently transfected with HA-tagged wild-type C99 (first row), or transiently co-transfected with GFP-tagged wild-type C83 and mCherry-tagged wild-type C99 (second row). After 16 h, cells were fixed and analyzed by fluorescence microscopy. H4 cells expressing HA-tagged C99 were permeabilized and double-labeled with mouse monoclonal antibody to HA, and sheep antibody to TGN46, followed by Alexa-498-conjugated donkey anti-mouse IgG (green channel), and Alexa-594-conjugated donkey anti-sheep IgG (red channel). Merging of the images in the green and red channels generated the third picture in the first and second row; yellow indicates overlapping localization of the green and red channels. Bar, 10 µm.
(TIF)

Figure S3 **Differential responses of wild-type APP and its CTFs to CQ and MG132.** H4 cells transiently expressing untagged, wild-type APP were left untreated or treated with 1 µM DAPT for 16 h, followed by either 100 µM CQ or 1 µM MG132 for 4 h in the absence or presence of 1 µM DAPT. Cellular extracts were analyzed by immunoblot with a rabbit polyclonal antibody raised against the cytosolic tail of APP. Immunoblot with anti-β-actin antibody was used as loading control. The positions of molecular mass markers are indicated on the left.
(TIF)

Figure S4 **Similar response of C99 and C83 to MG132 and CQ.** (A–B) H4 cells transiently expressing HA-tagged, wild-type C99 (A) or HA-tagged, wild-type C83 (B) were left untreated or treated for 16 h either with 100 µM CQ, 1 µM MG132 or with a combination of 100 µM CQ and 1 µM MG132. Cellular extracts were analyzed by immunoblot with mouse monoclonal antibody to HA. Immunoblot with anti-β-actin antibody was used as loading control. The positions of molecular mass markers are indicated on the left.
(TIF)

Figure S5 **Accumulation of C99 in the endoplasmic reticulum in response to treatment with BFA and MG132.** Confocal fluorescence microscopy of H4 cells stably expressing GFP-tagged C99 treated for 1 h with 5 µg/ml BFA alone or in combination with 1 µM MG132. Bars, 10 µm.
(TIF)

Figure S6 **Localization of C99 at the Golgi is enhanced in the absence of its ubiquitination.** H4 cells stably expressing GFP-tagged C99 or C99-5K/R were fixed, permeabilized, and labeled with sheep antibody to TGN46, followed by Alexa-594- conjugated donkey anti-sheep IgG (red channel). Stained cells were examined by confocal fluorescence microscopy. Merging of the images in the green and red channels generated the third picture in the first and second row; yellow indicates overlapping localization of the green and red channels. Bar, 10 µm.
(TIF)

Materials S1 **Supplemental information on plasmids and antibodies.**
(DOCX)
