# Supplementary figures and images for: Turnover of C99 is Controlled by a Crosstalk between ERAD and Ubiquitin-Independent Lysosomal Degradation in Human Neuroglioma Cells
Source: PLoS One. 2013 Dec 20;8(12):e83096. doi: 10.1371/journal.pone.0083096 (PMC3869756; doi:10.1371/journal.pone.0083096)

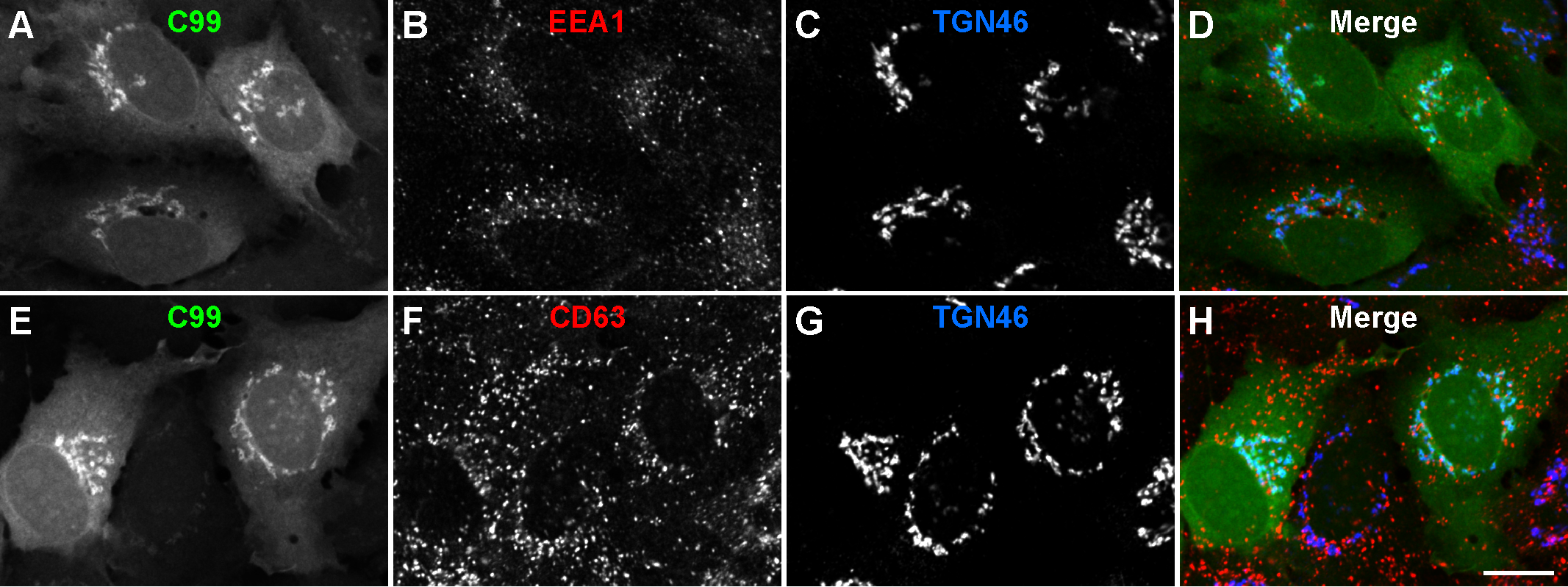

Supplement: Figure S1 — Intracellular localization of C99-GFP. H4 cells transiently expressing GFP-tagged C99 grown on coverslips were fixed, permeabilized, and double-labeled with mouse monoclonal antibodies to EEA1 or CD63, and sheep antibody to TGN46, followed by Alexa-594-conjugated donkey anti-mouse IgG (red channel), and Alexa-647-conjugated donkey anti-sheep IgG (blue channel). Stained cells were examined by confocal fluorescence microscopy. Merging of the images in the green, red, and blue channels generated the fourth picture in the first and second row; yellow indicates overlapping localization of the green and red channels, cyan indicates overlapping localization of the green and blue channels, magenta indicates overlapping localization of the red and blue channels, and white indicates overlapping localization of the red, green, and blue channels. Bar, 10 µm. (TIF) [file pone.0083096.s001.tif]

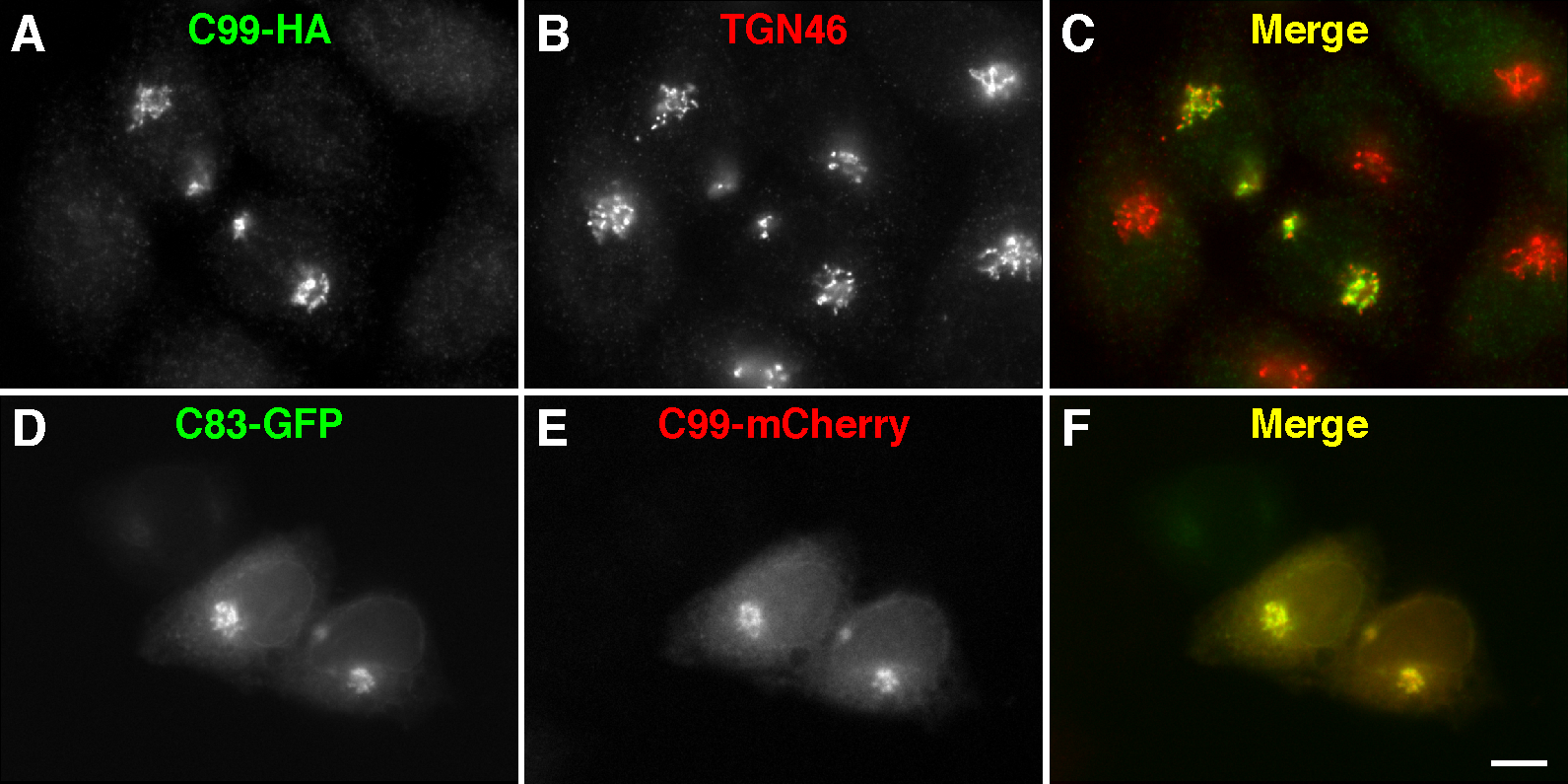

Supplement: Figure S2 — Intracellular localization of epitope-tagged C99 and C83. H4 cells grown on coverslips were transiently transfected with HA-tagged wild-type C99 (first row), or transiently co-transfected with GFP-tagged wild-type C83 and mCherry-tagged wild-type C99 (second row). After 16 h, cells were fixed and analyzed by fluorescence microscopy. H4 cells expressing HA-tagged C99 were permeabilized and double-labeled with mouse monoclonal antibody to HA, and sheep antibody to TGN46, followed by Alexa-498-conjugated donkey anti-mouse IgG (green channel), and Alexa-594-conjugated donkey anti-sheep IgG (red channel). Merging of the images in the green and red channels generated the third picture in the first and second row; yellow indicates overlapping localization of the green and red channels. Bar, 10 µm. (TIF) [file pone.0083096.s002.tif]

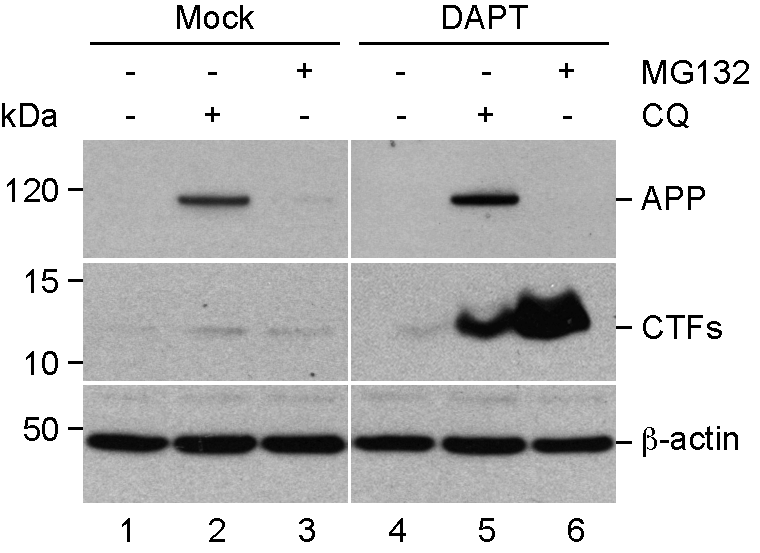

Supplement: Figure S3 — Differential responses of wild-type APP and its CTFs to CQ and MG132. H4 cells transiently expressing untagged, wild-type APP were left untreated or treated with 1 µM DAPT for 16 h, followed by either 100 µM CQ or 1 µM MG132 for 4 h in the absence or presence of 1 µM DAPT. Cellular extracts were analyzed by immunoblot with a rabbit polyclonal antibody raised against the cytosolic tail of APP. Immunoblot with anti-β-actin antibody was used as loading control. The positions of molecular mass markers are indicated on the left. (TIF) [file pone.0083096.s003.tif]

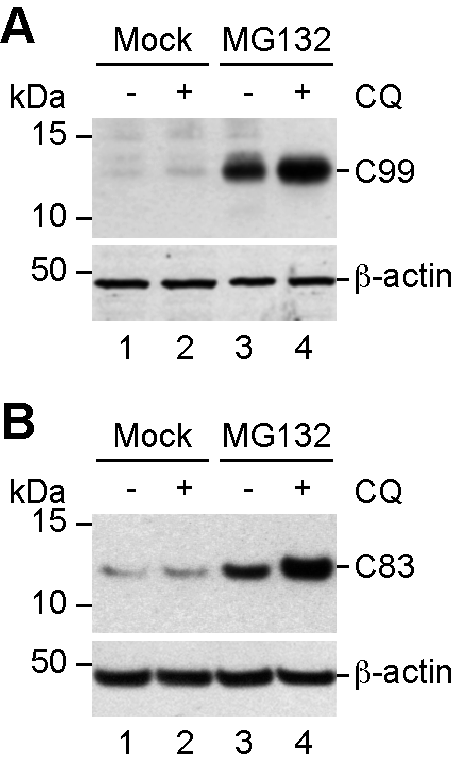

Supplement: Figure S4 — Similar response of C99 and C83 to MG132 and CQ. (A–B) H4 cells transiently expressing HA-tagged, wild-type C99 (A) or HA-tagged, wild-type C83 (B) were left untreated or treated for 16 h either with 100 µM CQ, 1 µM MG132 or with a combination of 100 µM CQ and 1 µM MG132. Cellular extracts were analyzed by immunoblot with mouse monoclonal antibody to HA. Immunoblot with anti-β-actin antibody was used as loading control. The positions of molecular mass markers are indicated on the left. (TIF) [file pone.0083096.s004.tif]

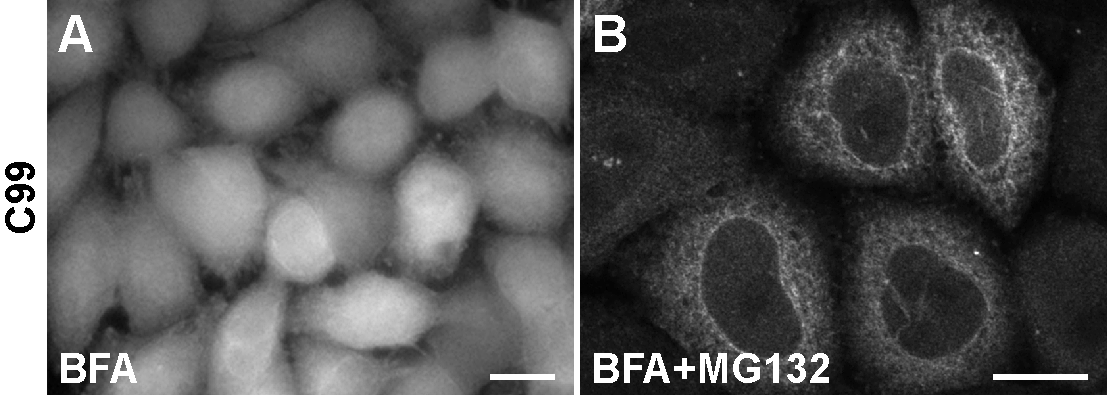

Supplement: Figure S5 — Accumulation of C99 in the endoplasmic reticulum in response to treatment with BFA and MG132. Confocal fluorescence microscopy of H4 cells stably expressing GFP-tagged C99 treated for 1 h with 5 µg/ml BFA alone or in combination with 1 µM MG132. Bars, 10 µm. (TIF) [file pone.0083096.s005.tif]

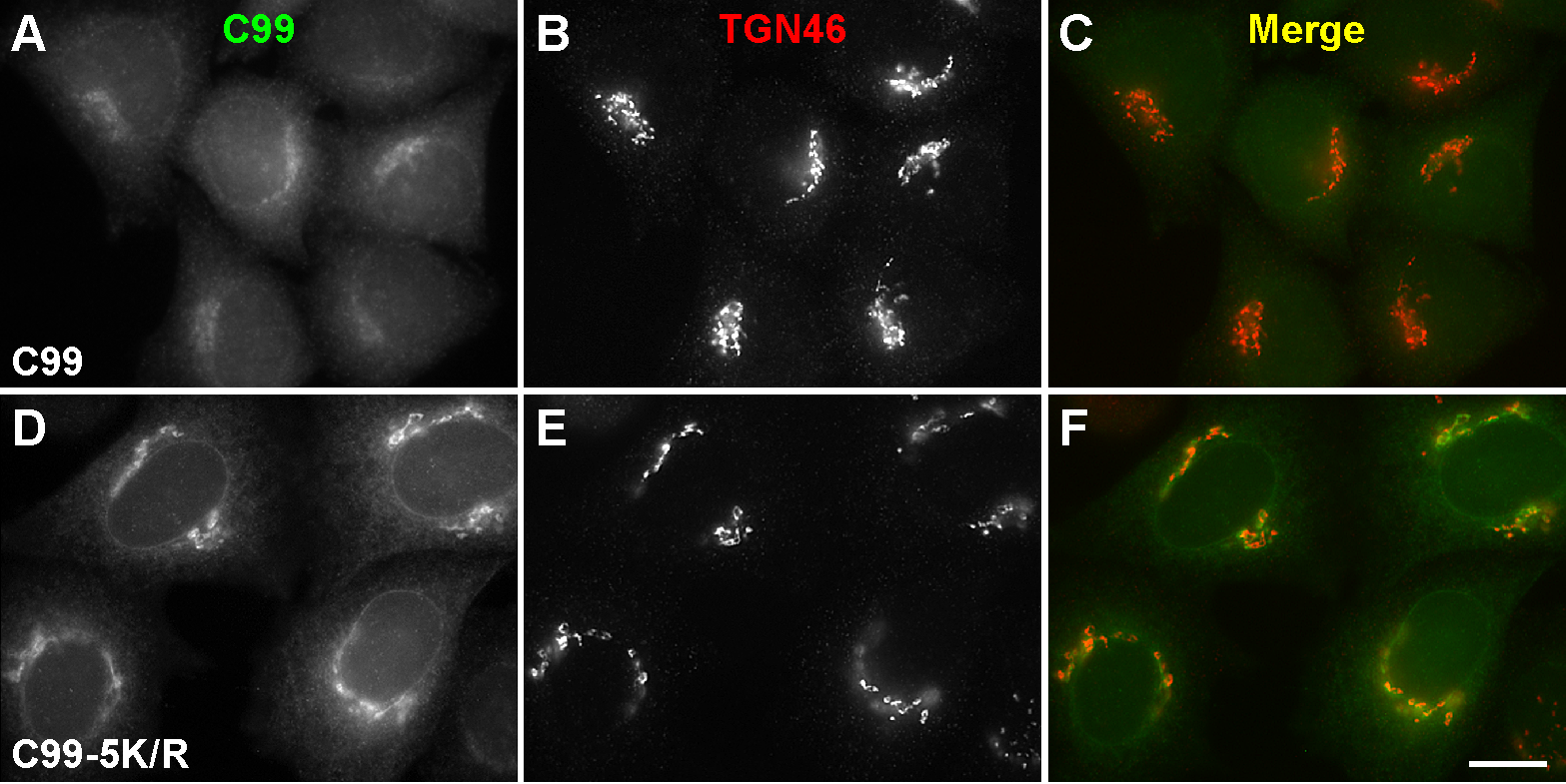

Supplement: Figure S6 — Localization of C99 at the Golgi is enhanced in the absence of its ubiquitination. H4 cells stably expressing GFP-tagged C99 or C99-5K/R were fixed, permeabilized, and labeled with sheep antibody to TGN46, followed by Alexa-594- conjugated donkey anti-sheep IgG (red channel). Stained cells were examined by confocal fluorescence microscopy. Merging of the images in the green and red channels generated the third picture in the first and second row; yellow indicates overlapping localization of the green and red channels. Bar, 10 µm. (TIF) [file pone.0083096.s006.tif]
