# Supplementary material for: Turnover of C99 is Controlled by a Crosstalk between ERAD and Ubiquitin-Independent Lysosomal Degradation in Human Neuroglioma Cells
Source: PLoS One. 2013 Dec 20;8(12):e83096. doi: 10.1371/journal.pone.0083096 (PMC3869756; doi:10.1371/journal.pone.0083096)
Supplement: Materials S1 — Supplemental information on plasmids and antibodies. (DOCX) [file pone.0083096.s007.docx]

**Materials S1.**

***Plasmids***

The constructs encoding wild-type, HA-tagged C99 or wild-type, GFP-tagged or HA-tagged C83, were generated using the QuickChange mutagenesis kit (Stratagene, La Jolla, CA). The C99-GFP construct was used as template to incorporate the sequence encoding a single HA tag (YDVPDYA) after the sequence encoding the last residue of C99, followed by a stop codon before the sequence encoding GFP. The GFP-tagged or HA-tagged C99 constructs were used as templates to generate wild-type, GFP-tagged or HA-tagged C83 constructs, respectively. The sequence encoding C83 (with an additional sequence encoding the signal peptide of APP) was generated with two consecutive rounds of QuickChange mutagenesis reactions; a mutagenesis reaction that deleted the first nine N-terminal residues of C99 (DAEFRHDSG), followed by a mutagenesis reaction that deleted the next ten N-terminal residues (YEVHHQKLVF). The mCherry-tagged C99 construct was generated by subcloning the sequence of C99 encoded in pEGFP-N1 into the *Eco*RI and *Sal*I sites of a plasmid encoding mCherry-N1. The construct encoding untagged, wild-type APP was generated by PCR amplification of the sequence encoding full-length, wild-type APP695 from the construct encoding APP-CFP described elsewhere (Burgos *et al.*, 2010), and cloned in-frame into the *Hin*dIII and *Sal*I sites of pEGFP-N1, with a stop codon after residue 695 before the sequence encoding GFP.

***Antibodies***

The following additional antibodies were used: rabbit polyclonal antibody to APP (CT695, Invitrogen), and mouse monoclonal antibody to HA (Covance, Dedham, MA).
